# Case report: Nocardial brain abscess in a persistently SARS-CoV-2 PCR positive patient with systemic lupus erythematosus

**DOI:** 10.3389/fmed.2022.973817

**Published:** 2022-08-18

**Authors:** Jelena Veličković, Tatjana Adžić Vukičević, Aleksandra Radovanović Spurnić, Igor Lazić, Bojana Popović, Ivan Bogdanović, Savo Raičević, Dragana Marić, Ivana Berisavac

**Affiliations:** ^1^COVID Hospital Batajnica, University Clinical Center of Serbia, Belgrade, Serbia; ^2^School of Medicine, University of Belgrade, Belgrade, Serbia; ^3^Clinic for Neurosurgery, University Clinical Center of Serbia, Belgrade, Serbia; ^4^Clinic for Pulmonology, University Clinical Center of Serbia, Belgrade, Serbia; ^5^Clinic for Neurology, University Clinical Center of Serbia, Belgrade, Serbia

**Keywords:** brain abscess, *Nocardia*, systemic lupus erythematosus, COVID-19, SARS-CoV-2, immunosuppression

## Abstract

Coronavirus disease (COVID-19) in immunocompromised patients represents a major challenge for diagnostics, surveillance, and treatment. Some individuals remain SARS-CoV-2 PCR-positive for a prolonged period. The clinical and epidemiological significance of this phenomenon is not well understood. We report a case of a patient with a history of systemic lupus erythematosus (SLE) who has been persistently SARS-CoV-2 PCR positive for 9 months, with multiple thromboembolic complications, and development of nocardial brain abscess successfully treated with surgery and antibiotics.

## Introduction

Coronavirus disease (COVID-19) caused by SARS-CoV-2 infection has elicited the ongoing pandemic with unprecedented impact on healthcare systems and over 6 million deaths by June 2022 (1). Evidence from well-designed clinical trials has enabled management for the majority of patients with COVID-19. However, conclusions from these trials may not apply to the cohort of immunocompromised patients in whom diagnostics, treatment, and surveillance still represent a significant challenge (2). Certain immunocompromised patients may develop persistent SARS-CoV-2 infection. The literature data regarding their potential for viral transmission, mutation accumulation, and possible further immunosuppression are scarce (3, 4). This patient cohort is also prone to developing opportunistic infections driven by chronic immunosuppressive therapy and persistent viral infection.

Nocardiosis is a rare infection in the form of a localized or disseminated disease caused by aerobic, weak acid-fast, branching bacilli of the *Nocardia* genus (5). They widely inhabit soil, water, and decomposing organic material and usually cause infection by inhalation or skin inoculation. Patients with cell-mediated immunodeficiency seem to be at higher risk (6). Nocardial brain abscess is exceptionally infrequent, making up around 2% of all brain abscesses (7). They can be misdiagnosed as tumors or brain metastases due to mass lesions seen on imaging, and a relatively silent clinical course. Despite the advancements in antimicrobial therapy, mortality of nocardial brain abscesses remains high and exceeds three times that associated with other bacterial pathogens (7).

We present a case of a patient with systemic lupus erythematosus (SLE) who has been persistently SARS-CoV-2 PCR positive for 9 months, with slow progression of COVID-19 with complications, and development of nocardial brain abscess successfully treated with surgery and antibiotics.

## Case report

**FIGURE 1:**
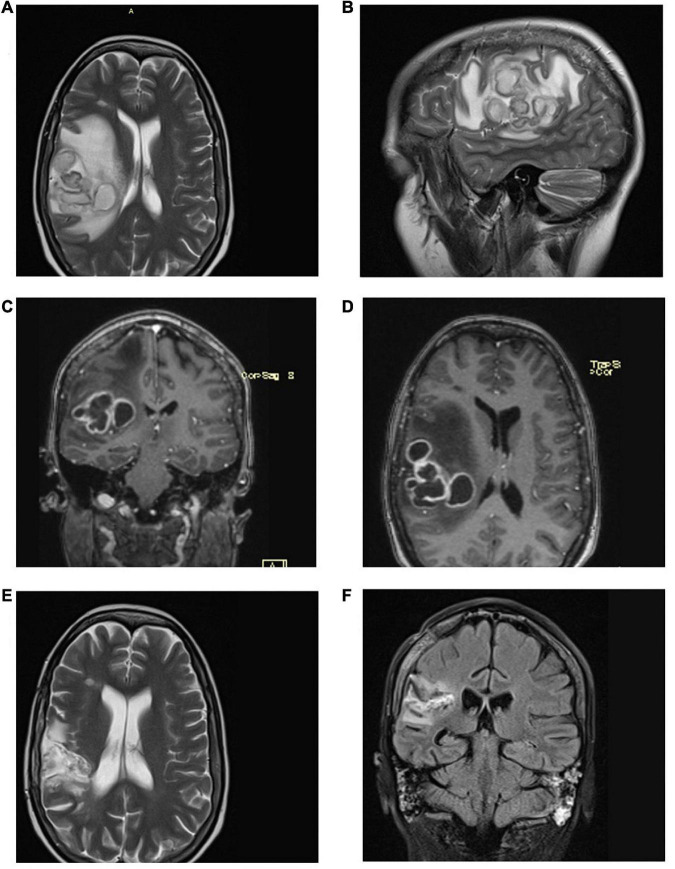
**(A,B)** Axial and sagittal plane of the brain MRI on June 15 shows a right parietal-temporal lesion with huge perilesional edema, compression of the right lateral ventricle, and mediosagittal shift; **(C,D)** Contrast-enhanced ring lesions on the coronal and axial plane with perilesional edema. **(E,F)** Postoperative MRI on September 1 with postoperative sequela, abscess regression, and perifocal edema.

**FIGURE 2:**
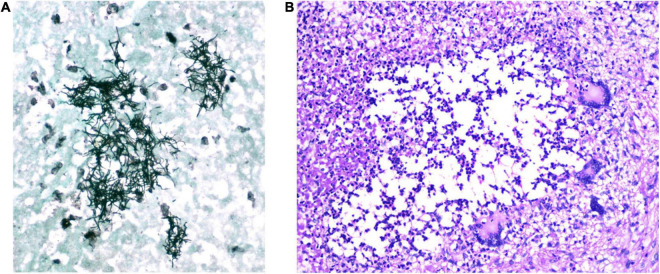
Patohistology finding of the material obtained on surgery **(A)** Grocott methamine silver staining shows branching microfilamentous bacilli of *Nocardia* spp. **(B)** Hematoxylin-eosin staining (magnification 200x): necrotizing granulomas of different sizes with the presence of multi-nuclear Langhans giant cells.

**FIGURE 3:**
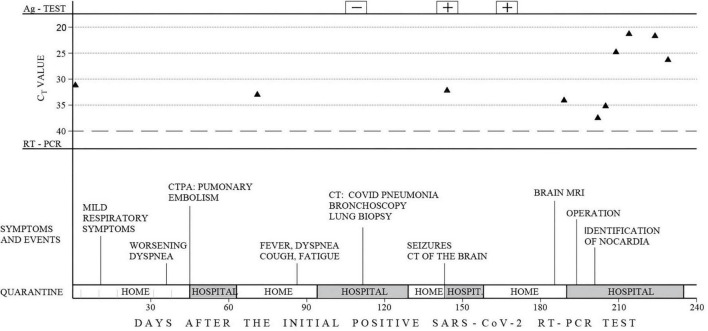
Timeline of the clinical course. Timelines of hospital admissions, symptoms, events and SARS-CoV-2 testing are labeled from the initial positive RT-PCR test. The Novel Coronavirus (2019-nCoV) Nucleic Acid Diagnostic Kit (PCR-Fluorescence Probing) for the qualitative detection of the ORF1ab and N genes of SARS-CoV-2 RNA was used with Ct value ≤ 40 considered a positive result.

On November 25, 2020, a 30-year-old woman with a 13-year history of systemic lupus erythematosus (SLE) exhibited painful leg swelling and was diagnosed with deep venous thrombosis (DVT). She was treated with enoxaparin, followed by an oral anticoagulant (warfarin). Her SLE initially presented as proteinuria, nephritis, and autoimmune hemolytic anemia was well-controlled and stable. She was treated during the last year with mycophenolate mofetil 250mg twice daily, prednisone 20mg/d, and hydroxychloroquine 200 mg/d. Several days following the development of DVT, the patient manifested symptoms of respiratory infection (subfebrile temperature up to 37.3°C, weakness, anosmia). She was tested (PCR of the nasopharyngeal swab) and found to be positive for SARS-CoV-2 on December 8. Due to the mild course of the disease, normal chest radiography, and good oxygen saturation of 99%, she was prescribed therapy and kept in home isolation. Nevertheless, the recovery from COVID-19 was not satisfactory as the patient complained of shortness of breath, persistent dull chest pain, extreme exhaustion, and tachycardia up to 130 bpm for weeks after the onset of the infection. She visited her immunologist on January 21, 2021. The laboratory analyses showed elevated inflammatory markers (CRP 27.5 mg/l [<5 mg/l]), d-dimer 3990 [<500], LDH 589 IU/l, WBC 8.3 × 10^9^/l, Lymphocyte 0.4 × 10^9^/l, and INR 1.93. Immunological analyses (Anti-nuclear antibodies -ANA, anti-Cardiolipin antibodies – aCLA IgM and IgG, anti-beta2 glycoprotein1 – anti-β_2_GPI IgM and IgG, lupus anticoagulant – LAC) were negative, total serum IgM and IgG were in the normal range, while total IgA was 0.62 g/l [0.8–2.4]. Computed tomographic pulmonary angiography (CTPA) demonstrated massive pulmonary embolism with the clot at the main branching of the pulmonary artery. She was hospitalized and treated until discharge home on February 8. Due to an allergy to warfarin, she has been prescribed rivaroxaban as further therapy. Two weeks following hospital discharge, the patient’s symptoms aggravated as she manifested morning fever up to 39°C, intense fatigue, dry cough, and chest pain. The persistence of these symptoms was the basis for admission to the pulmonology unit on March 12. On admission, she was slightly dyspneic with an oxygen saturation of 95% on room air. She had marked lymphopenia (0.3 × 10^9^/l), elevated CRP (91.9 mg/l), normal procalcitonin (0.05 ng/ml), and anemia (hemoglobin 91 g/L) with high ferritin value (856 μg/l). Initial chest CT scan demonstrated a multilocular irregular cystic formation (45 × 35 × 45 mm) in the right middle lobe adjacent to the visceral pleura, surrounded by a “tree in buds” appearance. It also showed ground-glass opacities (GGO) in both inferior lobes and the right middle lobe with the posterolateral and subpleural distribution. We performed ultrasound-guided lung biopsy to obtain samples to refer to microbiology and cytology analyses. They showed dense infiltration with lymphocytes, erythrocytes, and neutrophils, without malignant cells, while the microbiological culture showed no growth of microorganisms. Fiberoptic bronchoscopy revealed normal findings, and the specimen demonstrated rare neutrophils and *Pseudomonas spp.* (over 1 million CFU/mL) sensitive to amikacin. Hemoculture was negative, and the infection with *Mycobacterium tuberculosis* was excluded with smear acid-fast staining, PCR, MGIT culture test, Quantiferon TB Gold blood test, and negative Lowenstein culture growth. Galactomannan antigen (serum and bronchoalveolar) were negative, while serum mannan antigen was positive. The patient was initially treated with broad-spectrum antibiotics (Meropenem and Vancomycin), with the subsequent addition of amikacin and caspofungin. During hospitalization, the patient developed a cephalic vein thrombosis while on anticoagulant therapy, and she was thoroughly investigated for thrombophilia. Her antithrombin III, protein C, and protein S were in the normal range, and Anti-Cardiolipin antibodies (IgG, IgM, IgA) were negative. At the same time, the genetic screening for mutations (factor V Leiden, factor II G20210A, MTHFR, PAI-1) revealed no variants typical for inherited thrombophilia. The patient gradually recovered and was discharged home on April 15. On April 30, she had an episode of seizures with loss of consciousness. She was urgently admitted to the isolation unit of the neurology department of a regional hospital since she tested positive for SARS-CoV-2 again. Brain computed tomography (CT) revealed a solitary lesion with perifocal edema in the right parietal lobe. Antiedematous and anticonvulsant therapy diminished the neurological symptoms, and the patient was planned for referral to a tertiary center for further diagnostics and treatment. It was postponed as she tested positive again, and ordered home isolation. On June 15, the patient underwent brain magnetic resonance imaging (MRI). It showed a 4.6 × 4.3 × 3.9 cm multilocular lesion in the right frontoparietal region with a mass effect on the adjacent lateral ventricle (Figure 1). She tested positive for SARS-CoV-2 the same day and was denied admission to the neurosurgical clinic that offered no isolation ward. Instead, the patient was sent to a COVID hospital. At the time of admission, her symptoms were dry cough, stuffy nose, and mild left-sided weakness. Neurological examination showed subtle left side hemiparesis, right arm pronator drift, positive Mingazzini, and negative Romberg test. Laboratory workup revealed pancytopenia and normal tumor markers. Four days later, the neurological symptoms progressed with the development of holocranial headache, nausea, vomiting, and worsening of hemiparesis. An urgent CT of the brain was done, demonstrating enlargement of the perilesional edema with the shift of midline structures and completely effaced right lateral ventricle. A small lesion with perifocal edema was observed in the left occipital lobe. Right-sided compression of the mesencephalon suggested an imminent transtentorial uncal herniation. The patient was sent for emergency surgery on June 21 as she rapidly deteriorated with the development of a coma and right pupilar dilatation. The thick-walled multilocular abscess was found at surgery, and total excision was performed. Pus aspirated from one of the abscess collections was sent for gram staining and culture. The remaining was sent to histopathology. After the operation, the patient was transferred to the intensive care unit and started treatment with broad-spectrum antibiotics. Her postoperative laboratory was remarkable for severe thrombocytopenia, anemia, hypogammaglobulinemia, and low CD4 lymphocyte count (24/μL). Human immunodeficiency virus infection (HIV) was excluded by repeated testing. She was administered intravenous immunoglobulins (0.4 g/kg daily for 5 days). Hematoxylin-eosin staining revealed necrotizing granulomas, while the histochemical Grocott Methamine Silver staining highlighted the filamentous branching rods (Figure 2). Smears from cultures demonstrated gram-positive, partially acid-fast filaments, and the growth of *Nocardia* species. She was started on meropenem (3 × 2g) and trimethoprim/sulfamethoxazole (TPM/SMX; 960/4800 mg) *via* intravenous route while waiting for species determination and susceptibility testing. To transfer the patient to a non-COVID tertiary neurosurgical hospital, we tested her for SARS-CoV2 again, and the PCR result was positive. Final identification of *Nocardia cyriacigeorgica* was performed with matrix-assisted laser desorption ionization-time of flight mass spectrometry (MALDI-TOF MS). The susceptibility testing showed that the agent was susceptible to trimethoprim/sulfamethoxazole (TMP/SMX), ceftriaxone, cefotaxime, imipenem, and linezolid. The test demonstrated resistance to ampicillin, amoxicillin/clavulanate, and fluoroquinolones. TPM/SMX was continued as monotherapy after 3 weeks. Neurological deficits before surgery gradually resolved, and the CT of the brain showed significant improvement. Over the next 5 weeks, the patient was tested for SARS-CoV2 seven times, and the result was always positive (Figure 3). The serum sample was tested for antibodies against the spike protein of the virus, and no antibodies were detected. The patient was discharged home on July 30 on an oral TPM/SMX (160/800 mg) for at least 12 months. We decided to prescribe a prolonged course of TPM/SMX because her control CD4 was still low (79/μL), and she needed to continue corticosteroid therapy (prednisone 10mg/d) for SLE. On a follow-up as an outpatient after 1 month, 3 months, and 6 months the patient was in an excellent clinical state, fully active, with no symptoms and neurological deficits.

## Discussion

COVID-19 can be presented with a broad spectrum of clinical manifestations ranging from asymptomatic, mild, moderate, severe, to a clinical form requiring prolonged critical care (8). The early reports from Wuhan, China, demonstrated that the viral shedding in infected individuals is around 20 days, with the most extended observed duration of infectivity in survivors of 37 days (9). The study that differentiated viral dynamics in mild and severe cases of COVID-19 revealed that the mean viral load in severe cases was approximately 60 times higher (10). In contrast, the mild cases demonstrated an early viral clearance, with up to 90% of patients being tested negative on RT-PCR by day 10 (10). These studies were ground for recommendations from public health authorities in our country against routine PCR retesting after 14 days from the first positive test. Despite presenting initially with mild respiratory symptoms, our patient tested positive on RT-PCR of the nasopharyngeal swab for 233 days. An individual viral sequencing was unavailable in our country at the time of her treatment, so we couldn’t determine if she had re-infection with different strains of the SARS-CoV-2 virus. This is likely the longest PCR positivity reported in an adult patient. There is much uncertainty in the literature regarding the significance of post-symptomatic SARS-CoV-2 PCR positivity. It is unclear whether these patients carry transmission risk and whether they should be kept in self-isolation with limited access to non-urgent medical care. A study by Vibholm et al. conducted in a cohort of post-symptomatic COVID patients showed that 12.8% of patients remained PCR positive after 12 weeks following the first positive test (11). They found that the patients with the mildest disease, treated as outpatients with no limitations of daily activities, were more likely to stay persistently positive. Seroconversion did not affect viral shedding, and contact tracing revealed that persistent viral RNA detection was not associated with SARS-CoV-2 transmission (11). However, the population of immunocompromised patients was not enrolled in the study. The paper with the largest sample comparing retesting results in immunocompetent and immunocompromised patients revealed that solid organ transplant, older age, comorbidities (diabetes, obesity, and rheumatologic disease) were the risk factors for delayed SARS-CoV-2 PCR clearance, with no observed difference by the degree of immunocompromise (12). Nevertheless, several case reports demonstrated that immunocompromised individuals exhibit prolonged infectious SARS-CoV-2 shedding (3). Even more, during the prolonged course of the infection, virus evolution develops within the host with the emergence of mutations in the spike gene (3, 13). The important concern in persistent viral RNA carriers is the potential ensuing immunosuppression and its clinical consequences (14). It was daring in this case since the patient needed to continue her immunosuppressive therapy to control the underlying SLE.

Symptoms our patient experienced following acute SARS-CoV-2 infection, such as fatigue, shortness of breath, intermittent fever, and cough, resemble those described under the post-COVID-19 condition (15). It has been demonstrated that among various multiorgan symptoms of post-acute COVID-19 thromboembolic events occur in less than 5% of survivors (16). Our patient suffered several thromboembolic episodes, including massive pulmonary embolism. The last one ensued 113 days after the first positive RT-PCR test. The suspected antiphospholipid syndrome within SLE could not be confirmed since the patient didn’t meet the revised Sapporo criteria on several occasions (17). We assume that the prolonged hyperinflammatory state caused by persistent viral infection and systemic nocardiosis could have been responsible for the occurrence of thrombotic complications (18). Therefore, we prescribed acenocoumarol as an anticoagulation therapy after hospital discharge.

Systemic nocardiosis superimposed on COVID-19 has been previously described in a diabetic patient (19). To our knowledge, this is the first reported case of primary cerebral nocardiosis in a patient with persistent SARS-CoV-2 infection and SLE. Systemic infection with *Nocardia* spp. is most commonly found in immunocompromised patients, particularly those with depressed cell-mediated immunity, including solid organ and stem cell recipients, hematological malignancies, cancer patients receiving chemotherapy, patients with human immunodeficiency virus infection, and those on long-term corticosteroid treatment (5, 20). It has been shown in a murine model that both components of host defense contribute after the acquisition of nocardial infection (21). Neutrophils inhibit the growth of *Nocardia*, whereas cell-mediated immunity (CMI) is essential for the effective eradication of *Nocardia* in the first days following infection (21). The inhibition of CMI with Cyclosporine A and cortisone led to the formation of lung abscesses (21). Primary pulmonary nocardiosis is the most common clinical form of infection due to the mechanism of acquisition of infection by inhalation (22). Haemathologic spread may lead to the dissemination of infection to other sites, such as the skin, subcutaneous tissue, and central nervous system. Nocardial brain abscess is a severe clinical manifestation from the spectrum of central nervous system nocardiosis, with the highest mortality reaching over 35% (7). Since there is a paucity of clinical signs, no serological or biochemical markers of infection, and the radiologic imaging techniques are non-specific, awareness is fundamental for the diagnosis of nocardiosis (23, 24). Radiological features of solitary or multifocal ring-enhancing lesions are commonly misdiagnosed as brain metastases, tuberculosis, or bacterial abscess of other etiology (25).

Our patient with a nocardial brain abscess presented with overt immunosuppression. It has been shown that corticosteroid therapy represents the most important risk factor for the development of nocardial infection (26). However, the impact of persistent infection with SARS-CoV-2 on further immunosuppression can’t be neglected. It has been previously demonstrated that due to the down-regulation of various proteins associated with immune function, the immune system is suppressed early during COVID-19 (27). A robust CD4 T-cell response is crucial for the appropriate control of the disease (27). An extremely low CD4 count in our patient may explain an ineffective clearance of SARS-CoV-2, and failure to protect against the opportunistic infection with *Nocardia*. Although nocardial brain abscess is commonly secondary to the primary pulmonary site of infection, we couldn’t identify the primary focus in our patient despite a thorough investigation. We assume that the primary infection was subclinical, overshadowed by the CT feature of COVID-19 pneumonia, and masked by a short course of antibiotics that the patient received during the previous hospitalization (28).

Diagnosis and treatment of nocardial brain abscesses continue to be challenging. Due to the rarity of this clinical entity, no strict recommendations exist. Still, it seems that a more aggressive surgical approach with total excision of the lesion rather than aspiration warrants more promising outcomes (29, 30). Antimicrobial treatment should start with intravenous administration of at least two agents that represent the first-line therapy and includes TMP/SMX (31). Susceptibility testing is essential due to various resistance patterns, particularly with *Nocardia farcinica*. A recent study has shown a high sensitivity rate of *Nocardia* isolates to TMP/SMX (96.3%), amikacin (92.6%), and linezolid (100%). In contrast, the resistance rates were high to ceftazidime (69.3%), cefepime (64.5%), and amoxicillin/clavulanic acid (92.5%) (32). After a minimum of 3 weeks of intravenous therapy, patients can be switched to oral treatment. For patients with nocardiosis of the central nervous system, treatment should be continued for at least 12 months and even longer in immunocompromised patients who must be maintained on immunosuppressive therapy (24).

## Conclusion

We presented the case of an immunocompromised patient with COVID-19 and a persistent SARS-CoV-2 PCR positivity who developed a nocardial brain abscess during the course of the disease. Opportunistic central nervous system infections should be considered in the differential diagnosis of brain lesions in patients with immunosuppression. The inability to clear the virus in these patients may be a particularly important sign of suppressed immune systems. We believe that the good outcome in our patient was due to her young age, an aggressive surgical treatment followed by an adequate antimicrobial and other supportive therapy.

## Data availability statement

The original contributions presented in the study are included in the article/supplementary material, further inquiries can be directed to the corresponding author.

## Ethics statement

Written informed consent was obtained from the individual(s) for the publication of this case study. Written informed consent was obtained from the individual(s) for the publication of any potentially identifiable images or data included in this article.

## Author contributions

All authors listed have made a substantial, direct, and intellectual contribution to the work, and approved it for publication.
